# The effect of perioperative of dexamethasone on postoperative complications after pancreaticoduodenectomy (PANDEX): a study protocol for a pragmatic multicenter randomized controlled trial

**DOI:** 10.1186/s13063-023-07571-y

**Published:** 2023-09-02

**Authors:** Haoda Chen, Ying Wang, Chao Wang, Xiaojian Lu, Yilong Li, Bei Sun, Kuirong Jiang, Yudong Qiu, Rufu Chen, Liping Cao, Shi Chen, Yan Luo, Baiyong Shen

**Affiliations:** 1 Department of General Surgery, Pancreatic Disease Center, Ruijin Hospital, Shanghai Jiao Tong University School of Medicine, 197 Ruijin Er Road, Shanghai, 200025 China; 2 Department of Anesthesiology, Ruijin Hospital, Shanghai Jiao Tong University School of Medicine, 197 Ruijin Er Road, Shanghai, 200025 China; 3 Department of Pancreatic and Biliary Surgery, The First Affiliated Hospital of Harbin Medical University, Harbin, China; 4 Key Laboratory of Hepatosplenic Surgery (Ministry of Education), The First Affiliated Hospital of Harbin Medical University, Harbin, China; 5 Pancreas Center, The First Affiliated Hospital of Nanjing Medical University, Nanjing, China; 6 Department of Biliary and Pancreatic Surgery, Nanjing Drum Tower Hospital, The Affiliated Hospital of Nanjing Medical University, Nanjing, China; 7 Department of Pancreatic Surgery, Department of General Surgery, Guangdong Provincial People’s Hospital, Guangdong Academy of Medical Sciences, Guangzhou, China; 8 Department of General Surgery, Sir Run Run Shaw Hospital, School of Medicine, Zhejiang University, Hangzhou, China; 9 Shengli Clinical Medical College of Fujian Medical University, Fuzhou, China; 10 Department of Hepatobiliary Pancreatic Surgery, Fujian Provincial Hospital, Fuzhou, China

**Keywords:** Pancreaticoduodenectomy, Dexamethasone, Postoperative complications

## Abstract

**Background:**

Pancreaticoduodenectomy (PD) nowadays serves as a standard treatment for patients with disorders of the pancreas, intestine, and bile duct. Although the mortality rate of patients undergoing PD has decreased significantly, postoperative complication rates remain high. Dexamethasone, a synthetic glucocorticoid with potent anti-inflammatory and metabolic effects, has been proven to have a favorable effect on certain complications. However, the role it plays in post-pancreatectomy patients has not been systematically evaluated. The aim of this study is to assess the effect of dexamethasone on postoperative complications after PD.

**Methods:**

The PANDEX trial is an investigator-initiated, multicentric, prospective, randomized, double-blinded, placebo-control, pragmatic study. The trial is designed to enroll 300 patients who are going to receive elective PD. Patients will be randomized to receive 0.2 mg/kg dexamethasone or saline placebo, administered as an intravenous bolus within 5 min after induction of anesthesia. The primary outcome is the Comprehensive Complication Index (CCI) score within 30 days after the operation. The secondary outcomes include postoperative major complications (Clavien-Dindo≥3), postoperative pancreatic fistula (POPF), post-pancreatectomy acute pancreatitis (PPAP), infection, and unexpected relaparotomy, as well as postoperative length of stay, 30-day mortality, and 90-day mortality.

**Discussion:**

The PANDEX trial is the first randomized controlled trial concerning the effect of dexamethasone on postoperative complications of patients undergoing PD, with the hypothesis that the intraoperative use of dexamethasone can reduce the incidence of postoperative complications and improve short-term outcomes after PD. The results of the present study will guide the perioperative use of dexamethasone and help improve the clinical management of post-pancreatectomy patients.

**Trial registration:**

ClinicalTrials.gov NCT05567094. Registered on 30 September 30 2022

**Supplementary Information:**

The online version contains supplementary material available at 10.1186/s13063-023-07571-y.

## Background

Pancreaticoduodenectomy (PD)—also known as a Whipple procedure—is a complex operation that is used to treat tumors and other disorders of the pancreas, intestine, and bile duct. After the demanding resection, pancreatic-enteric, biliary-enteric, and gastrojejunal anastomoses are required to complete digestive tract reconstruction. Pancreatic-enteric anastomosis has remained a true Achilles’ heel in this complex and technically demanding operation. Despite improvements in operative techniques and perioperative management, the incidence of postoperative complications after PD remains high [1–3]. The main postoperative complications include postoperative pancreatic fistula (POPF), post-pancreatectomy hemorrhage (PPH), and delayed gastric emptying (DGE).

In recent years, post-pancreatectomy acute pancreatitis (PPAP) has been recognized as an independent complication after pancreatic resections [4–7]. Major surgery induces both local and systemic inflammation. A series of events related to operative trauma, ranging from manipulation, mobilization, alteration of blood supply, and/or stasis of pancreatic juice, appear to play a fundamental role in the etiology of PPAP [4, 8, 9]. These pathophysiological events may trigger a series of cascading events causing acinar cell disruption, intracellular activation of proteolytic enzymes, pancreatic parenchymal edema, and peripancreatic inflammation, leading to local and/or systemic effects [10]. Therefore, it is assumed that this inflammatory cascade of the remnant pancreas in the early postoperative reaction could be the possible mediator that triggers further downstream PD-related complications, particularly POPF and DGE [8, 11–14].

Dexamethasone is a synthetic glucocorticoid with potent anti-inflammatory and metabolic effects. In addition, a low dose of dexamethasone is frequently administered in the perioperative period, most commonly for prophylaxis of postoperative nausea and vomiting [15]. The mechanism of dexamethasone is poorly understood, but it seems to be most effective when it is administered before the start of surgery, as it can also reduce surgery-induced inflammation [16]. It is assumed that the antiemetic effect of dexamethasone is possibly related to its anti-inflammatory actions in the gastrointestinal tract.

Prolonged use of steroids such as dexamethasone can have dangerous side effects, including an increased risk of wound infection and anastomotic leak, which adversely affect recovery from gastrointestinal surgery. Although a single dose does not seem to be associated with these increased risks, it is reported that a single dose of dexamethasone was associated with hyperglycemia in surgical patients [17–19]. Though the DREAMS trial demonstrated that a single dose of 8 mg dexamethasone at induction of anesthesia could reduce nausea and vomiting rate after gastrointestinal surgery [20], concerns about the side effect among the surgical community might still be limiting its use during abdominal surgery. The PADDI trial confirmed that 8 mg dexamethasone was non-inferior to placebo with respect to the risk of surgical-site infection [19]. But few pancreatic surgeries are included in this study. Because PD consists of three anastomoses, the failure of which could result in dreaded complications, perioperative administration of dexamethasone has not been widely implemented into clinical practice in the setting of PD surgery. Recently, the Finland RCT reported that perioperative hydrocortisone significantly reduced postoperative complications after PD in high-risk patients [14]. In addition, PPAP is a series of early postoperative inflammatory reactions caused by the remnant pancreas. If the inflammation could be diminished, then the incidence of postoperative complications can be effectively reduced. Therefore, this study aims to assess the effect of dexamethasone on postoperative complications after PD.

## Methods/design

### Study design

**Fig. 1:**
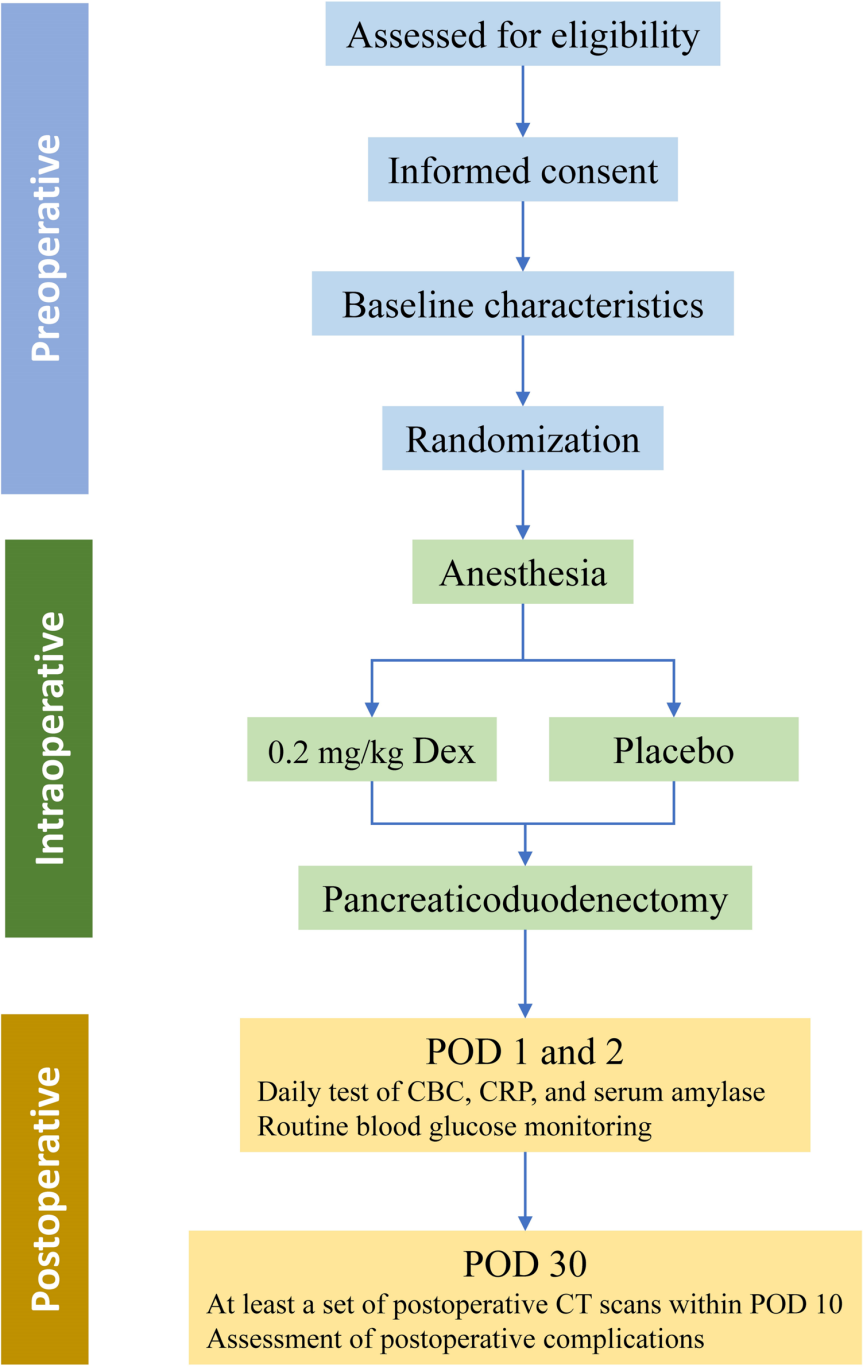
Study flowchart. Dex, dexamethasone; POD, postoperative day; CBC, complete blood count; CRP, C-reactive protein; CT, computed tomography

The PANDEX trial is an investigator-initiated, multicentric, prospective, randomized, double-blinded, placebo-control, pragmatic study. The trial will be conducted in seven high-volume centers in China (>50 PDs per year). This study hypothesizes that the intraoperative use of dexamethasone can reduce the incidence of postoperative complications after PD. The flowchart of this study is shown in Fig. 1. This article adheres to the Standard Protocol Items: Recommendations for Interventional Trials (SPIRIT) statement [21, 22].

### Participants

Patients who are going to receive elective PD in the participating centers will be reviewed for eligibility. A screening form will be used to exclude those who do not meet the following criteria. The inclusion criteria were as follows: (1) adult patients ≥18 years of age, (2) an indication for elective PD surgery, (3) patients understand the nature of this trial and are willing to comply, and (4) patients are able to provide written informed consent. The exclusion criteria were as follows: (1) current or recent (within preceding 1 month) systemic use of glucocorticoids; (2) distant metastases including peritoneal carcinomatosis, liver metastases, distant lymph node metastases, and involvement of other organs; (3) palliative surgery; (4) patients with high operative risk, as defined by the American Society of Anesthesiologists (ASA), with a score ≥ 4; (5) synchronous malignancy in other organs or second cancer requiring resection during the same procedure; (6) pregnancy; and (7) enrolled in other trials.

### Participant enrollment and withdrawal

Eligible patients will be assessed and informed by the specific study coordinators at the in-patient department. Recruited patients will be informed further in detail and will sign a written informed consent prior to treatment initiation, which can be revoked at any time. The duration of the recruitment period is estimated to be an 18-month interval depending on the recruiting rate. No financial incentives will be provided to the trial investigators or patients for enrolment during the recruitment period. All the patients will be free to participate in this study and can decide to withdraw at any time. If a patient withdraws, his/her information will not be recorded in the study.

### Randomization

After enrollment, randomization and allocation will be done on the day of surgery. The randomization will be centralized in the Guancui EDC system. We applied a randomized block design with a block number of six in a 1:1 ratio. A data manager who is independent of the data analysis or patient enrolment will generate the randomization schedule at the beginning of this trial. After providing informed consent, patients who are consistent with the inclusion and exclusion criteria will be randomly assigned to the treatment group or the control group. The randomization schedule will not be available to study recruiters or physicians. The allocation is only available for the independent data manager and the specific study coordinators in the Guancui EDC system. Specifically, when appropriate patients are enrolled, the researcher will inform the data manager, and then the random number and exact treatment group will be returned simultaneously.

### Blinding

The patients, surgeons, anesthetists, data collectors, and outcome assessors are all blinded. Only the data manager and the specific study coordinator are unblinded to the group allocation. Unblinding by the investigator for a given patient will occur only if the knowledge of treatment assignment will materially change the planned management of a medical emergency. Investigators will have direct access to a given patient’s individual study treatment and the decision to unblind a patient’s treatment assignment in an emergency situation resides with the investigator; however, the investigator should attempt to contact the medical monitor or appropriate study personnel to discuss options before unblinding the patient’s treatment assignment unless the urgency of the medical situation precludes this.

### Interventions

Patients will be randomized to receive 0.2 mg/kg dexamethasone or saline placebo, administered as an intravenous bolus within 5 min after induction of anesthesia. All other aspects of perioperative management will follow the regular protocols in our institution, as detailed in another trial protocol [23]. Non-trial glucocorticoids were prohibited for up to 7 days after surgery.

### Primary outcome measures

The primary endpoint is the Comprehensive Complication Index (CCI) score within 30 days after the operation. The CCI takes into account all cumulative complications and receives values between 0 and 100 (Table 1) [24]. The CCI’s weight of complication (wC) is based on the established Clavien-Dindo classification [25]. Detail calculation was presented below.

**Table 1:** The comprehensive complication index calculation

**Grade**	**wC**	**CCI single value**
Grade I	300	8.7
Grade II	1750	20.9
Grade III		
Grade IIIa	2750	26.2
Grade IIIb	4550	33.7
Grade IV		
Grade IVa	7200	42.4
Grade IVb	8550	46.2

Clavien-Dindo grade V always results in CCI 100

$CCI=\sqrt{wC1+wC2\dots +wCx}/2$; the online CCI calculator is available at www.assessurgery.com

*wC*, weight of complication; *CCI*, the Comprehensive Complication Index

### Secondary outcomes measures

The secondary endpoints are listed as follows:

1. The incidence of major complications (Clavien-Dindo≥3), 30 days
2. The incidence of postoperative pancreatic fistula (ISGPS classification), 30 days
3. The incidence of post-pancreatectomy acute pancreatitis (ISGPS classification), 30 days
4. The incidence of infection (including wound infection and intra-abdominal abscess), 30 days
5. The incidence of relaparotomy, 30 days
6. Postoperative length of stay
7. Mortality, 30 days
8. Mortality, 90 days

### Tertiary outcomes measures

The tertiary endpoints are listed as follows:

1. Serum CRP value on POD1 and POD 2
2. Average blood glucose levels on POD 1 and POD 2
3. Lymphocyte and neutrophil levels on POD 1 and POD 2
4. Overall medical costs

### Data collection and follow-up

All data will be collected after the patients provide informed consent. Patients will be followed according to normal postoperative routine in our institution. There is no extra outpatient clinic visit outside normal routine follow-up. All relevant factors will be recorded on a trial electronic case report form (eCRF). The schedule of enrolment, interventions, and assessments is shown in Table 2.

**Table 2:** Schedule for enrollment, interventions, and assessment

Timepoint**	**Study period**					
	**Enrolment**	**Allocation**	**Post-allocation**			**Close-out**
	***Preoperative*** ***visit*** ***-t***	***0***	***POD 0*** ***t1***	***POD 1*** ***t2***	***POD 2*** ***t3***	***POD 30*** ***tx***
**Enrolment:**						
**Eligibility screen**	X					
**Informed consent**	X					
**Allocation**		X				
**Interventions:**						
***Dexamethasone***			X			
***Saline placebo***			X			
**Assessments:**						
***Intraoperative outcomes***			X			
***Blood tests***				X	X	
***Primary/secondary outcomes***						X

*POD* Postoperative day

### Data management and monitor

In this study, the Guancui EDC system will be used for data collection. Patient data will be documented by dedicated trial personnel and managed in the eCRF. Data will be entered through a dedicated WeChat applet or web browser input masks. All staff involved in the data collection process are to be qualified to access the research database. Data collection must be collected in accordance with standard specification processes. The system cannot automatically identify the data needed to enter through manual input by the data entry person. All study-related information and participant information will be stored securely at the study site with locked cabinets in areas with limited access. The database will be secured with a password-protected access system.

An independent Data and Safety Monitoring Committee (DSMC) will be appointed to evaluate the study safety parameters. The DSMC will exist of two independent statisticians and two independent surgeons. DSMC members will meet before trial initiation, 3 months after the inclusion of the first patient, thereafter every 6 months, and after the interim analysis. All adverse events will be recorded on the eCRF. If the DSMC identify safety issues, they will have the prerogative to request unblinded outcomes. The safety of this trial will be evaluated in standardized intervals by the appointed institutional ethics and DSMC, which will advise regarding the trial’s status. More specifically, 30-day mortality will be monitored independently in each arm, and if a safety threshold of 5% is reached at any given time, the trial will be withheld for further safety evaluation and potential decision for early termination.

### Statistical analysis

#### Sample size calculation

The Finland RCT reported that perioperative hydrocortisone significantly reduced postoperative complications after PD in high-risk patients (18% vs. 41%; *P* < 0.05; Clavien-Dindo III-IV) [14]. Based on the pancreatic transection line frozen samples, high-risk patients with >40% acini consisted of more than half of the cohort. Because 0.2 mg/kg dexamethasone has theoretically a similar anti-inflammatory effect to hydrocortisone, so we presume that the perioperative dexamethasone could reduce at least 30% of major complications in the whole PD cohort.

For the sample size calculation, a 30% reduction in the rate of overall complications was assumed with a power of 80% (*β*=0·2), and an α-error of 0.05. This yielded an estimated group size of 128 patients. Finally, the sample size of this study is based on the primary outcome — CCI. Because CCI is more sensitive and requires a smaller sample size compared to the Clavien-Dindo classification in RCT, 128 patients in each group are sufficient for the primary outcomes [24]. Finally, the target recruitment of the PANDEX trial will be set at 300 to account for 20% dropout or deviation of the protocol. Thus, 150 patients are required in each group.

#### General analysis principle

Normality will be examined using the Shapiro-Wilk test. For normal distribution, continuous variables will be described as mean and standard deviation (SD) and compared using the *t*-test. For non-normal distribution, continuous variables will be described as medians and interquartile range (IQR) and compared using the Mann-Whitney test. test. Categorical variables will be expressed as frequencies and percentages and compared using chi-squared test or Fisher exact test when appropriate. We have no imputation plans for missing data. All statistical tests will be tested with two sides, *P* < 0.05 is considered to represent a statistically significant difference, with a CI of 95%. All analyses were performed using Stata (Stata Corporation, version 15, College Station, TX, USA).

#### Efficacy analysis

All outcomes will be analyzed using modified intention-to-treat analyses, where all randomized patients who undergo scheduled pancreaticoduodenectomy were included in the analyses. Criteria to remove patients from analyses after randomization are as follows:

- Scheduled pancreaticoduodenectomy was not performed (e.g., disseminated cancer, enucleation).
- Total pancreatectomy was performed.
- Additional hepatic resection.
- Patient withdrew consent prior to surgery.

The primary outcome is a sum of all complications that are weighted for their severity, based on the Clavien-Dindo classification, the Comprehensive Complication Index (CCI). CCI score will be presented as mean ± SD and in case of skewed distributions as median and range. Values will be compared by the *t*-test or the Mann-Whitney test as appropriate.

#### Interim analysis

Planned interim analyses will be performed by the DSMC at recruitment of 150 patients. Any recommendation to proceed with the trial or to abandon for either safety or futility reasons will be communicated with the principal investigators in the first instance. The DSMC is the final arbitrator as to the necessity for safety reporting to the regulatory authorities and/or individual sites.

#### Subgroup analysis

Exploratory post-hoc subgroup analyses are considered to further explore the effects of dexamethasone on postoperative complications. For example, subgroup analyses can be stratified as follows:

- Robotic pancreaticoduodenectomy vs. open pancreaticoduodenectomy
- Patients with pancreatic duct adenocarcinoma vs. with other diseases
- Soft pancreatic texture vs. hard pancreatic texture
- Preoperative diabetes vs. no diabetes

## Discussion

With the development of surgical techniques, the high incidences of postoperative complications after PD become the greatest challenge for pancreatic surgeons [26, 27]. To date, few trials have reported satisfactory results. So, there is an urgent need to find effective management to reduce the morbidities after PD.

A single low-dose dexamethasone before the operation is a simple treatment for anesthetists but could make real changes in the postoperative course of PD patients. The anti-inflammatory effect of dexamethasone would mitigate PPAP and reduce the incidence of postoperative complications after PD. The PANDEX trial will test this hypothesis in a high-volume pancreatic center. The results might provide us with a potentially effective therapy for the routine practice of pancreatic surgery and alleviate the burden of postoperative complications in PD patients.

## Trial status

This trial was registered at ClinicalTrials. gov (NCT05567094) on September 30, 2022. Patient recruitment was initiated in September 2022 and is estimated to be completed by October 2023. At the time of submission this trial has recruited 120 patients. This article is based on the most recent version of the study protocol (version 2.1, May 2023). Any modifications of the protocol will be updated at ClinicalTrials. gov.

### Supplementary Information

**Additional file 1.** SPIRIT Checklist for Trials. 

## Data Availability

Not applicable.

## Abbreviations

CBC: Complete blood count
CCI: Comprehensive Complication Index
CRP: C-reactive protein
DSMC: The Data and Safety Monitoring Committee
eCRF: Electronic case report form
EDC: Electronic Data Capture
PD: Pancreaticoduodenectomy
POD: Postoperative day
